# Acral melanoma presenting with multiple intussusceptions: a case report and literature review

**DOI:** 10.3389/fonc.2026.1793853

**Published:** 2026-03-30

**Authors:** Guoqiang Zhang, Jiangang Tang

**Affiliations:** Shengzhou People’s Hospital (Shengzhou Branch of the First Afliated Hospital of Zhejiang University School of Medicine, the Shengzhou Hospital of Shaoxing University), Shengzhou People’s Hospital, Shaoxing, China

**Keywords:** acral melanoma (AM), brain metastasis, intussusception, KIT, multi-disciplinary team (MDT)

## Abstract

Acral melanoma (AM) is a distinct subtype of melanoma unrelated to ultraviolet exposure, and its amelanotic variant presents considerable diagnostic challenges due to its occult clinical manifestations. Here, we report a rare case of amelanotic acral melanoma (AAM) with brain metastasis, initially presenting as multiple small intestinal intussusceptions. A 66-year-old male presented with abdominal pain and intestinal obstruction. Imaging revealed two sites of small bowel intussusception and space-occupying lesions in the brain. Emergency laparoscopic resection of three intestinal tumors was performed, and postoperative pathological examination confirmed metastatic melanoma. Positron emission tomography-computed tomography (PET-CT) suggested a suspicious primary lesion at the distal end of the right first metatarsal. Genetic testing revealed an activating KIT p.L576P mutation. Following multidisciplinary team discussion, the patient received palliative whole-brain radiotherapy combined with oral imatinib targeted therapy. One month after treatment, neurological function improved significantly, and imaging showed marked regression of the brain metastases. Three months post-treatment, follow-up PET-CT revealed no Fluorodeoxyglucose (FDG) uptake at the distal right first metatarsal. This case highlights the following: (1) Metastatic melanoma should be considered in the differential diagnosis of unexplained adult intussusception; (2) PET-CT is of significant value in detecting occult primary lesions; (3) Genetic testing should be performed in patients with acral melanoma to guide targeted therapy; (4) A multidisciplinary integrated diagnosis and treatment model combining local intervention for acute complications with systemic targeted therapy represents an effective strategy for advanced complex cases.

## 1 Introduction

Acral melanoma (AM) is a distinct melanoma subtype unrelated to ultraviolet exposure, with an incompletely understood pathogenesis. It accounts for 2%–3% of cutaneous melanomas but has a higher incidence in Asian populations (1). AM typically arises on the palms, soles, and subungual regions (2). Due to concealed locations, atypical presentations, and high heterogeneity, diagnosis is often delayed, with most patients presenting at advanced stages (III–IV), leading to poor prognosis (3). A comprehensive overview of current knowledge on melanoma diagnosis and treatment has been provided by Caraviello et al. (4).

Amelanotic Acral melanoma (AAM) is rarer, often lacking typical pigmentation, and is frequently identified initially due to metastatic symptoms (5). Common metastatic sites include the lungs, brain, and abdominal organs. While intestinal metastasis in melanoma can reach 60%, presentation with intussusception as the first symptom is extremely rare, and most reported cases have identifiable pigmented primary lesions (6). No systematic report has described AAM with brain metastasis initially presenting as multiple intussusceptions. We report a 66-year-old male with multiple intussusceptions ultimately diagnosed as AAM with brain metastasis, discussing clinical characteristics, diagnostic approaches, and individualized treatment strategies based on genetic testing.

This study was approved by the Institutional Ethics Committee, and informed consent was obtained. This case is reported in accordance with SCARE guidelines (7).

## 2 Case presentation

### 2.1 Initial presentation (Day 0)

**Figure 1:**
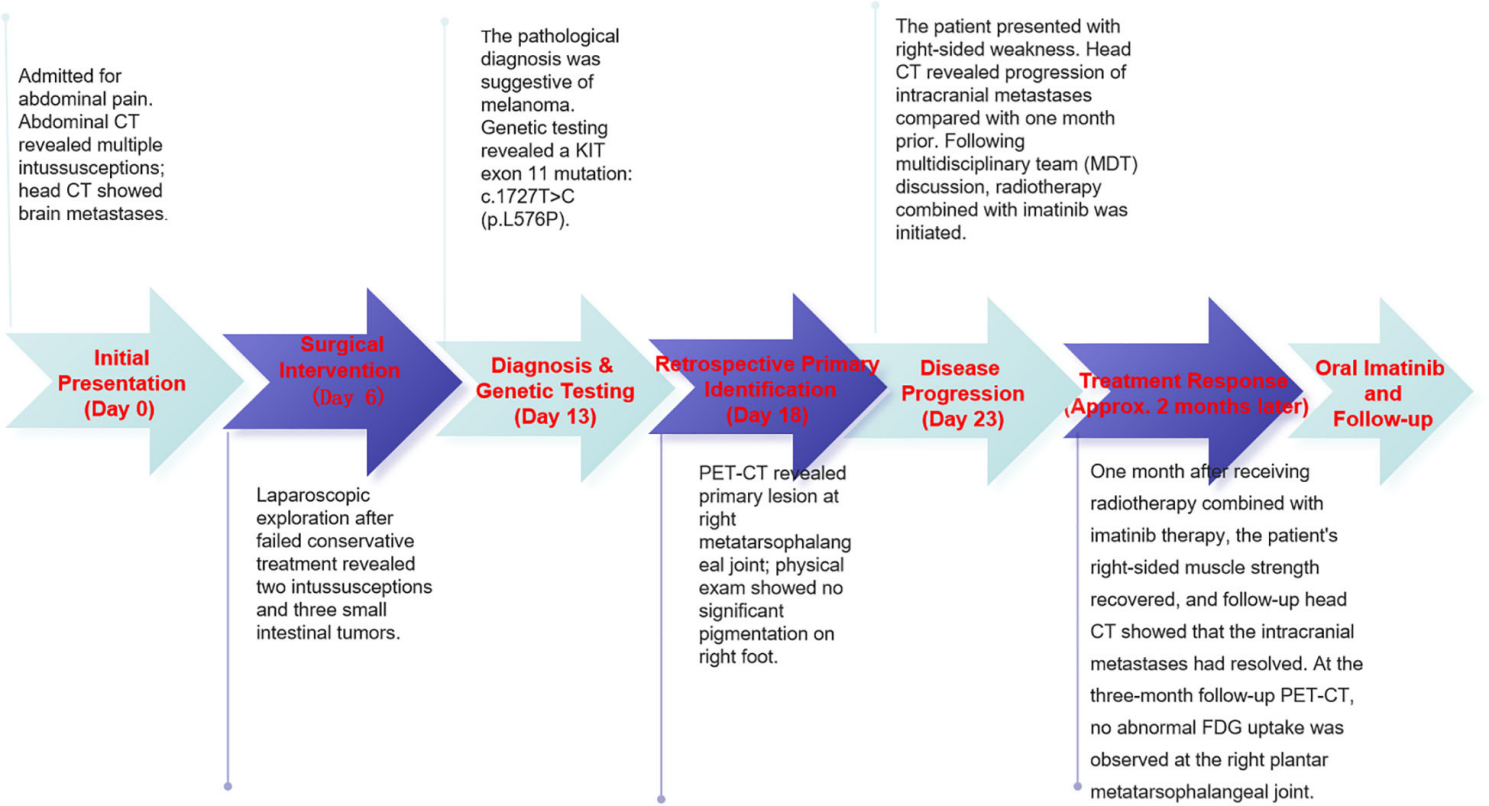
Timeline of patient admission, diagnosis, and clinical management.

The patient (**Figure 1**), a 66-year-old male, was admitted to the hospital with a chief complaint of intermittent dull pain and abdominal distension for half a month. The abdominal pain could be slightly relieved after passing gas or having a bowel movement. During the course of illness, there was no hematochezia, nausea, vomiting, or fever. Past medical history revealed no previous surgeries, trauma, or drug allergies. Family history was negative for tumors or genetic diseases.

Physical examination on admission: No jaundice or signs of anemia were observed over the skin and mucous membranes. No palpable enlargement of superficial lymph nodes. The abdomen was flat, with mild tenderness in the left lower quadrant, but no rebound tenderness or muscle guarding. No definite mass was palpated. Tympany was noted on percussion, and bowel sounds were approximately 5 per minute.

**Figure 2:**
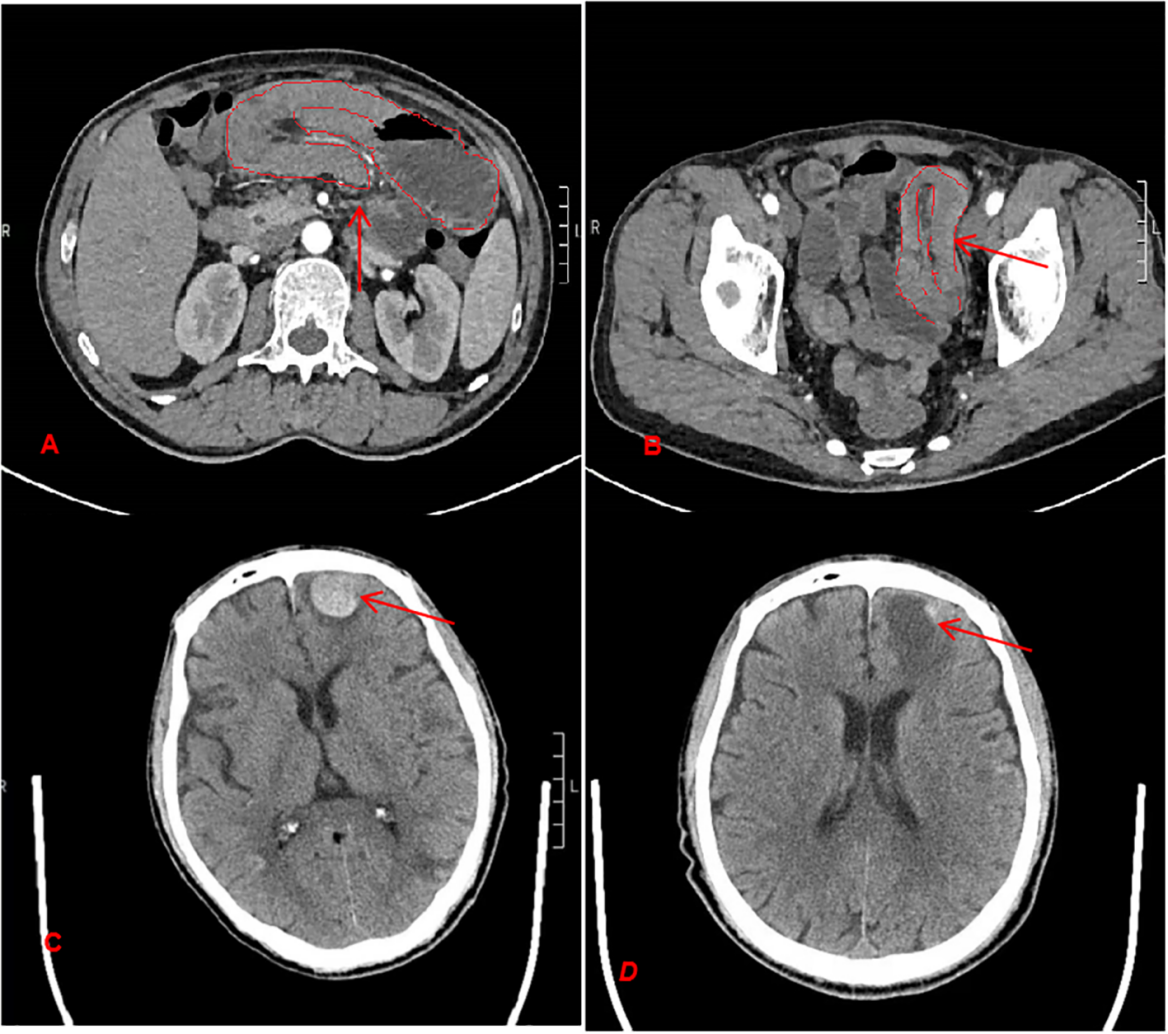
CT findings of small bowel intussusception and intracranial metastases. **(A, B)** Contrast-enhanced abdominal CT scans showing small bowel intussusception. The arrows indicate the characteristics target sign **(A)** and the intussusception bowel loop at the site of intussusception **(B, C)** Pre-treatment head CT showing a metastatic lesion in the left parietal lobe, with local hyperdensity (arrow). **(D)** Post-radiotherapy head CT demonstrating near-complete resolution of the previously seen metastasis (arrow), with mild post-treatment edema in the adjacent brain parenchyma.

Auxiliary examinations: Abdominal Computed Tomography (CT) revealed two sites of small bowel intussusception with incomplete intestinal obstruction (**Figures 2A, B**). Tumor markers (AFP, CEA, CA125, CA199, CA153) were all within normal limits.

Diagnosis and treatment process: Upon admission, conservative treatments including antispasmodics and fluid replacement were administered, but the patient continued to experience intermittent abdominal pain. Preoperative workup, including a head CT, revealed an intracranial space-occupying lesion, suspected to be a metastatic tumor (**Figure 2C**).

### 2.2 Surgical intervention (Day 6)

**Figure 3:**
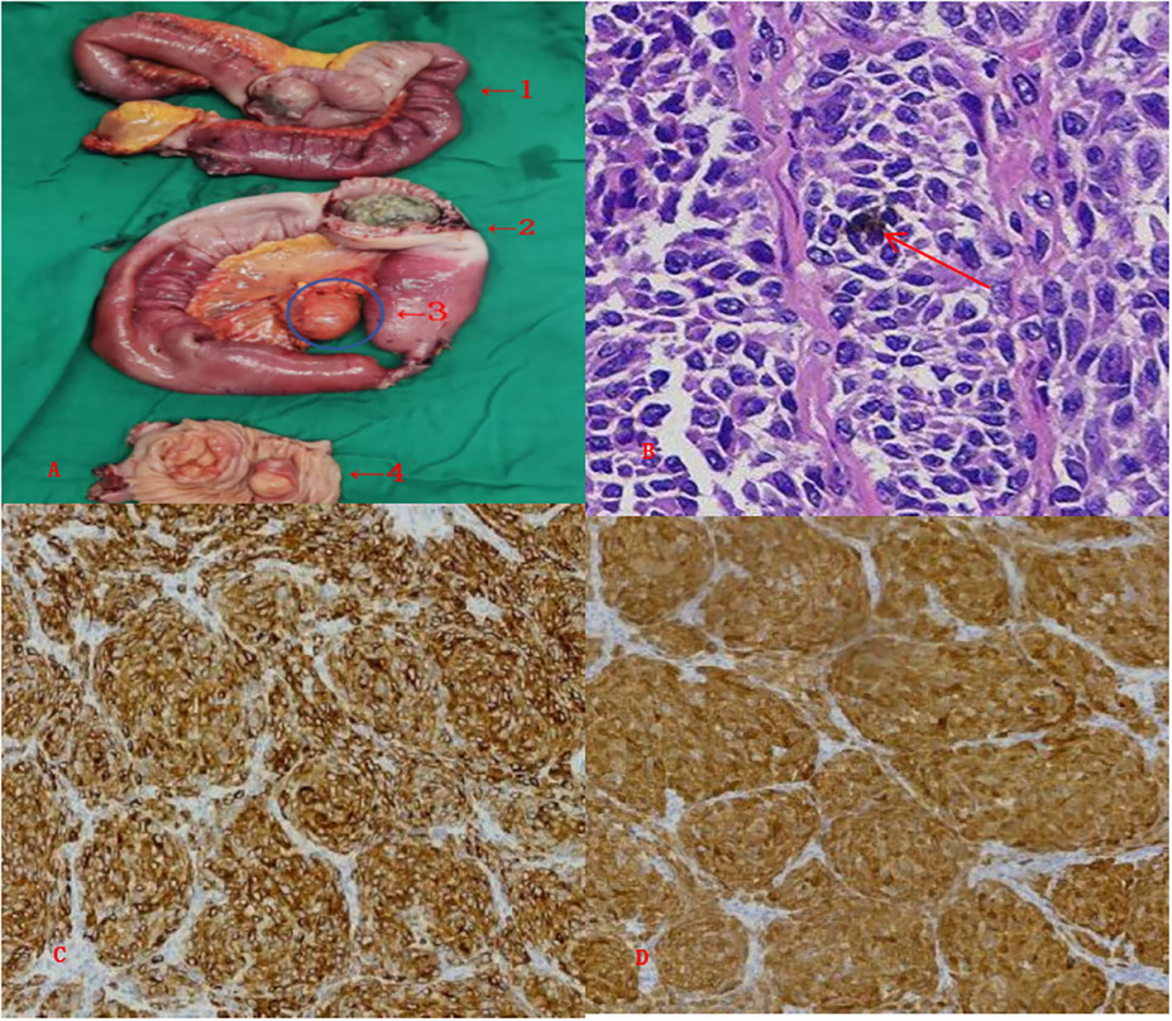
Pathological features of intestinal metastatic tumors. **(A)** Gross examination: The submitted specimen includes two segments of ileum (1 and 2) and one segment of jejunum (3), with intraluminal metastatic tumors observed in all segments. Intussusception is present in the ileum (1 and 2), and the intussuscepted bowel wall appears pale due to ischemia. An enlarged regional lymph node (4) is noted adjacent to the ileum (2). Two metastatic lesions are identified within the jejunum (3), with the cut surface of the tumors appearing pinkish and soft in texture. **(B)** Histopathological image (hematoxylin and eosin staining, X 100): High-power magnification reveals abundant brown-black pigment granules within the nuclei of tumor cells (red arrow). **(C, D)** Immunohistochemical staining ( X 100): The tumor cells show diffuse strong positive expression for Melan A **(C)** and S100 **(D)**, supporting the diagnosis of melanoma.

As the abdominal pain persisted without relief after conservative treatment, a laparoscopic exploration was performed. Intraoperatively, two sites of intussusception were found in the ileum, approximately 50 cm (Ileum 1) and 80 cm (Ileum 2) proximal to the ileocecal valve. Additionally, two firm tumors were palpated in the jejunum, about 40 cm distal to the ligament of Treitz. A segmental small bowel resection with side-to-side anastomosis was subsequently performed. Postoperative specimen examination is shown in **Figure 3A**.

### 2.3 Diagnosis and genetic testing (Day 13)

Postoperative pathological diagnosis: Pathological diagnosis revealed that all three intestinal tumors were malignant melanoma (microscopic features shown in **Figure 3B**). The immunophenotype was positive for Melan-A, S-100 (**Figures 3C, D**), HMB-45, and SOX10.

Genetic testing results: A KIT gene mutation in exon 11, c.1727T>C (p.L576P), was detected with a variant allele frequency of 52.28%, classified as a class I activating mutation. Additionally, a copy number loss of the CDKN2A gene (CN = 1.16) was identified.

### 2.4 Retrospective primary identification (Day 18)

**Figure 4:**
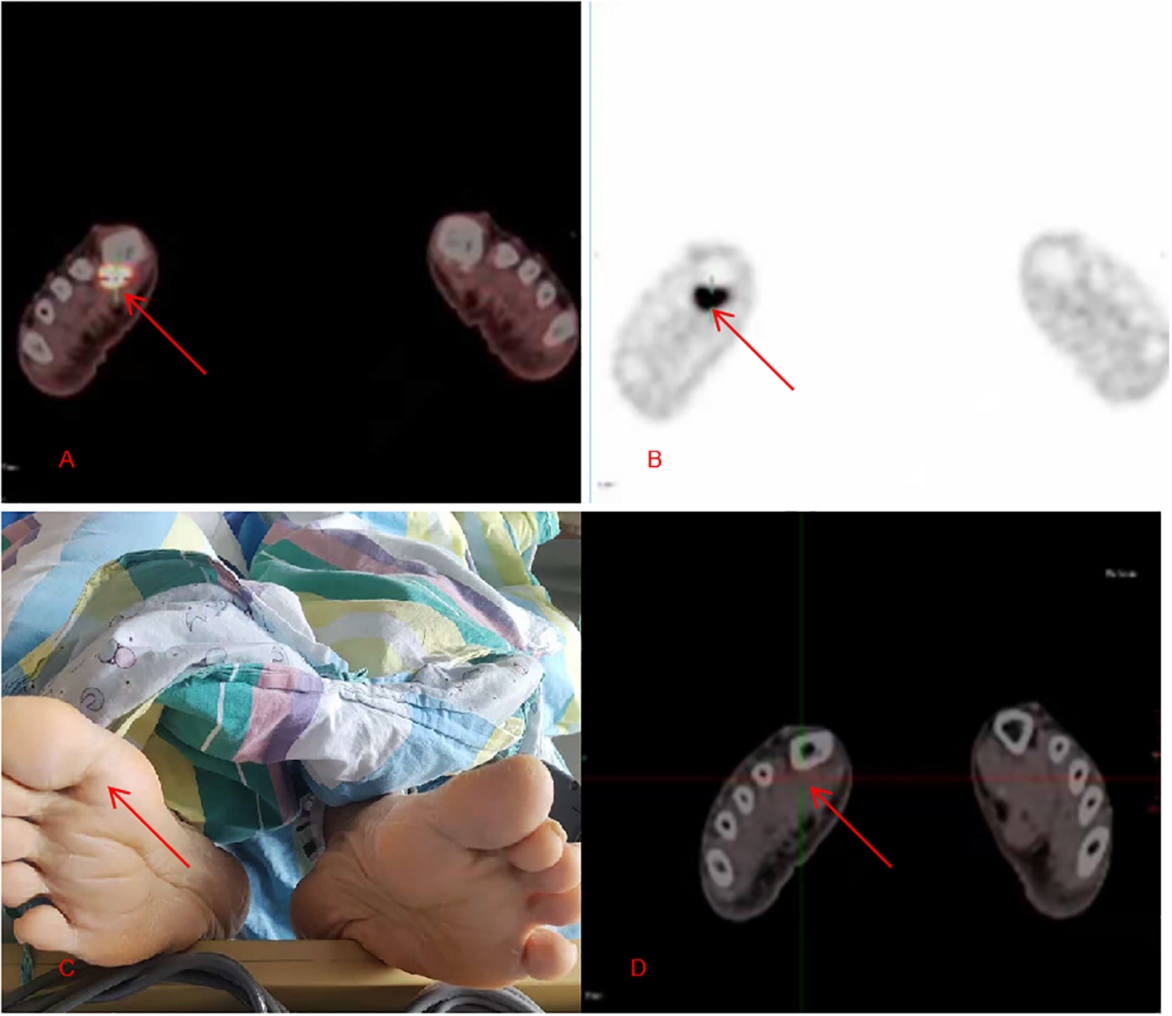
**(A, B)** (Pre-treatment): PET-CT shows an FOG-avid lesion at the right metatarsophalangeal joint (red arrow); **(C)** (Pre-treatment physical examination): No cutaneous pigmentation is observed at the right plantar metatarsophalangeal joint (red arrow); **(D)** (Post-treatment): PET-CT shows disappearance of the previously observed FOG-avid lesion at the right metatarsophalangeal joint (red arrow at the intersection of the red and green lines).

Positron Emission Tomography-Computed Tomography(PET-CT) revealed soft tissue thickening with increased Fluorodeoxyglucose(FDG) uptake at the distal end of the right first metatarsal bone (near the metatarsophalangeal joint), considered the primary melanoma lesion (**Figures 4A, B**). Nodular foci with high FDG metabolism were observed in the left cerebral hemisphere, consistent with metastatic lesions. Further inquiry into the medical history revealed no history of trauma to the right foot. Physical examination showed no deformities of the lower limbs and no pigmentation or obvious abnormalities on the right foot (**Figure 4C**).

### 2.5 Disease progression (Day 23)

The patient developed right-sided hemiplegia, with muscle strength declining to Medical Research Council Scale for Muscle Strength(MRC) grade 1 +. Following multidisciplinary team (MDT) discussion, an immediate decision was made to initiate palliative radiotherapy for the brain metastases and simultaneously start oral targeted therapy with Imatinib (400 mg once daily).

### 2.6 Disease progression and favorable response (Approx. 2 months later)

Following treatment, the patient demonstrated significant recovery of right-sided muscle strength to MRC grade 4+, and imaging evaluation confirmed marked regression of the brain metastases (**Figure 2D**). At the 3-month follow-up, PET-CT revealed no abnormal FDG uptake at the right plantar metatarsophalangeal joint (**Figure 4D**). The patient achieved complete resolution of gastrointestinal symptoms, including abdominal pain and nausea, resumed a normal diet, and regained independence in daily activities, reflecting substantial improvement in quality of life. He expressed great satisfaction with both the surgical outcome and the overall treatment process. The patient remains under regular follow-up and continues oral imatinib maintenance therapy.

## 3 Discussion

### 3.1 Intussusception as a rare initial presentation of melanoma

Amelanotic acral melanoma (AAM) progresses rapidly and portends a poor prognosis, often presenting initially with metastasis, with the brain, lungs, and gastrointestinal tract being common sites. Although gastrointestinal metastases are present in up to 60% of melanoma-related deaths, only 1.5%–4.4% are diagnosed antemortem, highlighting the insidious nature of its clinical manifestations (8). Adult intussusception is frequently secondary to organic lesions such as tumors (9), and surgery holds both diagnostic and therapeutic value. The mechanism often involves intramural tumors causing localized bowel wall rigidity, leading to invagination of the proximal segment into the affected area during peristalsis. While isolated case reports exist of small bowel intussusception caused by metastatic melanoma (10, 11), presentation with multiple synchronous intussusceptions, as seen in this case, necessitates expanding the differential diagnosis from common etiologies to rare ones. The patient’s age and the presence of multiple lesions strongly suggest malignancy; the multifocal nature particularly points towards metastatic disease—primary cancers are typically solitary, whereas metastases are often multiple.AAM presenting initially with multiple, simultaneous intussusceptions is exceedingly rare, suggesting that metastatic tumors, especially melanoma, should be carefully considered in the differential diagnosis of unexplained adult intussusception.

Furthermore, intestinal metastases from melanoma are frequently amelanotic (12), further complicating preoperative and intraoperative identification. Given that metastases are often multiple and the small bowel is highly mobile, meticulous laparoscopic exploration is recommended to identify the locations of intussusception and metastatic tumors. Conversion to open surgery may be necessary to perform a systematic examination of the entire bowel and avoid missed metastases. This strategy helps minimize incisions and reduce the risk of wound infection (13).

### 3.2 Concealed primary lesion and diagnostic challenges

In this case, the primary lesion was located at the metatarsophalangeal joint of the foot and was amelanotic. Due to its anatomically concealed location, it was easily misdiagnosed early on as a callus, plantar wart, or other benign condition, often leading to a delayed diagnosis until after metastasis has occurred. Dermoscopic evaluation, particularly using standardized scoring models such as the iDScore_plantar developed by Tognetti et al. (14), can aid in the early differentiation of acral melanoma from benign mimics on the soles.For metastatic melanoma of unknown primary, PET-CT holds critical diagnostic value (15). In this case, whole-body PET-CT screening, based on the hypermetabolic activity of tumor cells, successfully localized the primary lesion at the right first metatarsophalangeal joint while also comprehensively assessing the extent of metastases, providing a crucial foundation for accurate staging (Stage IV) and subsequent treatment decisions. Lens M et al. (8) also emphasized the important role of imaging in differentiating primary from secondary melanoma of the small intestine.

### 3.3 Precision therapy for KIT-mutant acral melanoma

The incidence of KIT mutations in acral and mucosal melanoma is approximately 10%–20% (16), among which hotspot mutations such as L576P and K642E are closely associated with tumor development, progression, and sensitivity to tyrosine kinase inhibitors (TKIs) (17). The patient in this case was diagnosed with stage IV melanoma (brain and multiple intestinal metastases), with limited expected efficacy from chemotherapy alone. Following guideline recommendations, genetic testing of the tumor tissue was performed, revealing a KIT p.L576P mutation. After treatment with radiotherapy combined with imatinib, the brain metastases showed significant regression within a short period, further confirming the high sensitivity of this mutation to TKIs and underscoring the necessity of genetic testing in guiding precision therapy for advanced melanoma. Additionally, concurrent CDKN2A copy number loss was detected in this case, which may synergistically promote tumor progression with the KIT pathway through dysregulation of the cell cycle, highlighting the complexity of the molecular mechanisms involved. Future therapeutic strategies should integrate multi-gene information to achieve stratified intervention.

### 3.4 Value of the multidisciplinary treatment model

This case illustrates an effective strategy integrating local and systemic therapies. Emergency surgery resolved the acute intussusception, enabling subsequent treatment. Precise radiotherapy controlled the symptomatic brain metastasis, providing a critical window for targeted therapy to take effect. Following multidisciplinary discussion, systemic treatment with radiotherapy (18) combined with oral imatinib achieved durable disease control. This multidisciplinary approach—coordinating local and systemic management for both acute and chronic conditions—represents an effective clinical pathway for advanced melanoma, particularly in patients with severe metastases or complications (19).

Due to practical clinical constraints, this study did not perform dermatoscopy or pathological biopsy on the suspicious lesion on the patient’s right sole (at the metatarsophalangeal joint). The identification of the primary lesion relied primarily on PET-CT imaging findings and the characteristics of the disease, which constitutes the main limitation in the chain of evidence for this study.

## 4 Conclusion and implications

This case provides key insights for the management of acral melanoma: (1) Atypical acute abdominal presentations in adults, such as intussusception, should prompt consideration of neoplastic causes, including metastatic melanoma, in the differential diagnosis. (2) In cases of metastasis from an unknown primary, comprehensive systemic imaging—such as PET-CT—should be performed to define the extent and origin of disease. (3) Routine genetic testing is recommended for specific subtypes, such as acral or mucosal melanoma, to identify driver mutations (e.g., KIT) and inform targeted therapy. (4) Patients with advanced-stage disease should be managed through a multidisciplinary team (MDT) approach, integrating local interventions (e.g., surgery, radiotherapy) with systemic targeted therapy. Addressing acute or critical lesions can create a window for subsequent systemic treatment, ultimately improving outcomes and prognosis.

## Data Availability

The original contributions presented in the study are included in the article/supplementary material. Further inquiries can be directed to the corresponding author.
